# Reference Values for Physical Stress Echocardiography in Asymptomatic Patients after Mitral Valve Repair

**DOI:** 10.3389/fsurg.2018.00006

**Published:** 2018-02-19

**Authors:** Rosemarijn Jansen, Kim Urgel, Maarten J. Cramer, Egidius E. H. L. van Aarnhem, Peter P. M. Zwetsloot, Pieter A. Doevendans, Jolanda Kluin, Steven A. J. Chamuleau

**Affiliations:** ^1^Department of Cardiology, University Medical Center Utrecht, Utrecht, Netherlands; ^2^Department of Cardiology, St Antonius Hospital Woerden, Woerden, Netherlands; ^3^Department of Cardiothoracic Surgery, University Medical Center Utrecht, Utrecht, Netherlands; ^4^Department of Cardiothoracic Surgery, Academic Medical Center Amsterdam, Amsterdam, Netherlands

**Keywords:** physical stress echocardiography, two-dimensional transthoracic echocardiography, mitral valve repair, mean transmitral pressure gradient, systolic pulmonary artery pressure

## Abstract

**Background:**

Clinical decision-making in symptomatic patients after mitral valve (MV) repair remains challenging as echocardiographic reference values are lacking. In native MV disease intervention is recommended for mean transmitral pressure gradient (TPG) >15 mmHg or systolic pulmonary artery pressure (SPAP) >60 mmHg at peak exercise. Insight into standard stress echo parameters after MV repair may therefore aid to clinical decision-making during follow-up.

**Hypothesis:**

Stress echocardiography derived parameters in asymptomatic patients after successful MV repair differ from current guidelines for native valves.

**Material and methods:**

In 25 patients (NYHA I) after MV repair stress echocardiography was performed on a semi-supine bicycle. Doppler flow records and MV related hemodynamics at rest and peak were obtained. Linear regression analysis was performed for mean TPG and SPAP at peak, using predetermined variables and confounders.

**Results:**

Mean TPG at rest (3.2 ± 1.4 mmHg) significantly increased at peak (15.0 ± 3.4 mmHg) but was always <25 mmHg. Mean SPAP at rest (21.4 ± 3.8 mmHg) significantly increased at peak (41.8 ± 8.9 mmHg) but was never >57 mmHg. Only the indexed MV ring diameter was inversely correlated to mean TPG at peak in a multivariable model.

**Conclusion:**

In contrast to current recommendations in native MV disease, our data indicate that the standard value for mean TPG during stress echocardiography in asymptomatic patients after successful MV repair was above the guideline threshold of 15 mmHg in >50%, but always <25 mmHg. For SPAP, patients never reached the guideline cutoff (60 mmHg). Long-term follow-up data are needed to provide insight in clinical consequences. Baseline stress echocardiography may indicate individual reference values to compare with during follow-up.

**Clinical trial registration:**

https://clinicaltrials.gov/ct2/show/NCT02371863?term=chamuleau+AND+Mitral&rank=1.

## Introduction

Mitral valve (MV) regurgitation is a common valve disease and associated with significant morbidity and mortality worldwide (1, 2). The etiology is classified as organic (primary) or functional (secondary). In organic MV regurgitation, a component of the MV apparatus is diseased, for which surgery (preferably MV repair) is the only approach with defined clinical success (3–7). The global rate of MV repair increased between 2000 and 2007 from 42 to 61% (8). The procedure comprises a correction of the abnormal apparatus, followed by remodeling of the MV annulus using a ring (2, 9). These restrictive techniques may lead to some degree of narrowing of the MV orifice. The resulting functional stenosis has been reported in the context of pannus overgrowth from the annuloplasty ring, and the use of a small and/or complete ring (10–14). Although high success rates and low mortality numbers are reported (15–18), recurrent or persistent complaints after isolated MV repair remain a clinical challenge, in particular when transthoracic echocardiography (TTE) parameters are within normal range at rest. In this respect, exercise echo parameters may be helpful by complementing resting two-dimensional (2D) TTE in clinical practice (19–22). However, current guidelines present only normal stress echo values for (1) asymptomatic patients with MV regurgitation (class IIb: surgery if systolic pulmonary artery pressure (SPAP) >60 mmHg at peak exercise) and (2) symptomatic MV stenosis (class IIb: percutaneous valvulotomy if SPAP > 60 mmHg or mean transmitral pressure gradient (TPG) >15 mmHg, including suitable MV morphology) (3, 4). Reference values in patients after successful MV repair are lacking, consequently it is uncertain if a TPG of >15 mmHg or SPAP > 60 mmHg in a symptomatic patient after MV repair can be used as a clinically useful parameter. This hampers the implementation of stress echocardiography in daily practice for this specific dilemma. Hence, the purpose of our study was to provide insight into standard stress echo derived parameters in asymptomatic patients after successful MV repair. We hypothesize that these values differ from current guidelines for native valves, which may aid to clinical decision-making at follow-up.

## Materials and Methods

Twenty-five randomly selected patients after successful isolated MV repair at the UMC Utrecht were analyzed by use of an echocardiographic assessment at rest and during exercise. The flowchart is depicted in Online Resource 1 in Supplementary Material. Successful MV repair was defined as no or grade 1+ residual of recurrent MV regurgitation and mean TPG ≤ 7 mmHg after MV repair in rest at time of study enrollment (4). Inclusion criteria were as follows: (1) age ≥18 years; (2) asymptomatic (NYHA class 1); (3) at least 6 months after successful MV repair, followed by a cardiac rehabilitation program with return to an active lifestyle; (4) sinus rhythm; (5) normal postoperative TTE at rest including SPAP < 50 mmHg, mean TPG < 5 mmHg and absence of concomitant valve disease of more than mild severity; and (6) no comorbidity that may hamper exercise testing (e.g., physical inability). Information on the MV etiology, pathology, and surgical techniques was derived from the surgical database. Postoperative follow-up data were requested from the treating physician. MV annuloplasty ring diameter was indexed for body surface area, as previous studies did for MV effective orifice area (23). The study protocol was approved by the Research Ethics Board, and informed consent was obtained from all patients. The primary objective was to determine mean TPG and SPAP at peak exercise in asymptomatic patients after successful MV repair. Second, we studied two subgroups based on the (1) mean TPG at peak exercise and (2) SPAP at peak exercise.

### Transthoracic Echocardiography

Echocardiographic measurements were obtained in accordance with current guidelines and the Declaration of Helsinki (24, 25). Images were performed on the Philips IE33 echo-machine (Philips Medical Systems, Andover, MA, USA) by an experienced imaging cardiologist. Datasets were archived according to local routine (Philips Xcelera software R3.3L1). Data analysis was done by two observers (Rosemarijn Jansen and Kim Urgel). Left ventricular (LV) dimensions, stroke volume, and LV ejection fraction (EF) were obtained at rest and peak exercise following the guidelines (24, 25). The MV regurgitation severity was graded 0–4 according to current recommendations, including grade 0.5 for trace severity. The peak and mean TPG were calculated based on the modified Bernoulli equation on the transmitral continuous-wave Doppler signal. SPAP was calculated using the modified Bernoulli equation on the transtricuspid continuous-wave Doppler signal, adding the estimated right atrial pressure. Change in SPAP was determined by subtraction of the SPAP at rest from the peak value. Since previous results showed that the pressure halftime method is not reliable and progressively overestimates the MVA, e.g., during exercise-induced heart rate changes, we determined MVA on the continuity equation (26).

### Physical Stress Testing

Patients underwent a physical exercise test on a semi-supine bicycle. We monitored a 4-lead electrocardiogram during exercise and measured blood pressure (non-invasively) before, during, and after exercise. Symptoms were continuously evaluated. A standard exercise protocol was used, starting with 25 W, which was manually increased by 25 W every 3 min. Echocardiographic recordings were performed at each stage. To determine echo values at peak, stress echocardiography was preferentially performed until >85% of the age-predicted maximal heart rate or exhaustion occurred. Medication was continued to assess exercise tolerance under baseline treatment.

### Statistical Analysis

Statistical analysis was done using SPSS (version 21.0, IBM Corporation, NY, USA). Continuous variables were expressed as mean ± SD and compared with a two-sided *T*-test for 2 groups, one-way ANOVA for >2 groups, or Mann–Whitney *U* test in case of non-normal distribution. Categorical data were described using frequencies and percentages, with comparative evaluations performed *via* the χ^2^, Fisher’s Exact, or McNemar’s test. A *P*-value of <0.05 was considered statistically significant. Linear regression for (1) mean TPG at peak and (2) SPAP at peak (continues values) was performed based on complete case analysis, with univariable and multivariable linear regression on predetermined variables of interest (MV annuloplasty ring type and indexed diameter, surgical resection performed, and SPAP and TPG related parameters, respectively) and potential confounders (age, gender, MVA at rest, MV regurgitation grade at peak, heart rate at peak, EF at rest, and total months after MV repair).

## Results

### Patient Characteristics

Baseline characteristics and preoperative profile are displayed in Table 1. Age ranged from 31 to 75 years. In all patients, the etiology was organic, including degenerative MV disease in 23 subjects (92%) and endocarditis in 2 (8%). Surgery was performed *via* median sternotomy, cardiopulmonary bypass, and cardioplegic arrest with cold blood cardioplegia. Quadrangular or triangular resection of the MV leaflet was performed in 17 (68%), annuloplasty using a Carpentier–Edwards Physio Ring in 15 (60%), and annuloplasty with a Cosgrove–Edwards Annuloplasty Band in 10 (40%) patients. A concomitant procedure, e.g., Maze or coronary bypass surgery, was never performed. The mean TPG measured by echocardiography within the first 5 days after repair was always <5 mmHg (3.1 ± 0.9 mmHg).

**Table 1:** Baseline characteristics (*n* = 25).

	Total population
**Patient characteristics**	

Age (mean, ±SD)	54.0 (9.5)
Male (*n*, %)	19 (76)
BSA, m^2^ (mean, ±SD)	2.0 (0.2)

Cardiac rhythm during stress echo (*n*, %)	
– Sinus rhythm – Atrial fibrillation	25 (100) 0 (0)

NYHA (*n*, %)	
Class I Class >I	25 (100) 0 (0)

Baseline medication (*n*, %)	
– Beta-blocker – ACE/ATII antagonist – Diuretics	3 (12) 5 (20) 0 (0)

Comorbidities (*n*, %)	
– COPD/asthmatic bronchitis – Chronic kidney disease – Prior PCI – Prior malignancy – Physical inability – Other	2 (8) 0 (0) 0 (0) 1 (4) 0 (0) 0 (0)

General condition/sportive state (*n*, %)	
– Good, frequent activities – Moderate, random activities – Poor, no activities	20 (80) 5 (20) 0 (0)

**Operation characteristics**	

Primary MV regurgitation etiology (*n*, %)	25 (100) 13 (52) 10 (40) 2 (8) 0 (0)
– Myxomatous degeneration – Fibroelastic degeneration – Endocarditis – Other	

Mitral leaflet pathology (*n*, %)	
– Anterior – Posterior – Both	2 (8) 22 (88) 1 (4)

MV surgery (*n*, %)	
– MV repair – Quadrangular or triangular resection – Neochordea – MV replacement	25 (100) 17 (68) 6 (24) 0 (0)

MV annuloplasty (*n*, %)	
– C–E Physio Ring 30/32/34/36/40 mm – Cosgrove–Edwards band 32/34/36 mm	1 (4)/4 (16)/6 (24)/3 (12)/1 (4) 3 (12)/3 (12)/4 (16)

Indexed MV annuloplasty ring diameter^a^ (mean, ±SD)	17.6 (2.0)
Time interval since surgery, months (mean, ±SD)	27 (16)

*BSA, body surface area; NYHA, New York Heart Association; COPD, chronic obstructive pulmonary disease; PCI, percutaneous coronary intervention; MV, mitral valve; C–E, Carpentier–Edwards*.

*^a^Indexed for BSA*.

### Resting Echocardiographic Data

Table 2 depicts data at rest and peak exercise, as measured by 2D TTE at 27 ± 16 months after MV repair. The LV dimensions were normal in all patients, whereas the LV function quality at rest was reduced in 16%. Resting mean TPG was always ≤7 mmHg. Two patients showed a baseline mean TPG of >5 mmHg (6.6 and 7.0 mmHg) despite a value of <5 mmHg directly post MV repair, and in absence of MV annuloplasty ring dysfunction on the baseline TTE. The heart rates at rest reached high-normal values (95 and 83 bpm, respectively). Significant residual or recurrent MV regurgitation was never seen. SPAP in rest was always <50 mmHg. Patients with no SPAP value due to the absence of tricuspid regurgitation, showed no other signs of elevated pulmonary pressures.

**Table 2:** Stress echocardiographic characteristics at rest and peak exercise (*n* = 25).

	Resting	Peak exercise	*P*-value	Change peakrest
Heart rate, bpm (mean, ±SD)	76 (10)	151 (15)	0.000	76 (16)
SBP, mmHg (mean, ±SD)	132 (18)	204 (27)	0.000	72 (19)
DBP, mmHg (mean, ±SD)	84 (9)	92 (13)	0.000	8 (7)
MAP, mmHg (mean, ±SD)	100 (11)	130 (15)	0.000	29 (8)
LV EF, % (mean, ±SD)	56.4 (4.9)	59.4 (5.5)	0.000	2.9 (2.1)
LV EF quality (*n*, %)			1.000	
– Good/impaired/poor	21 (84)/4 (16)/0 (0)	21 (84)/4 (16)/0 (0)		–
LVESD, mm (mean, ±SD)	3.6 (0.4)	–	–	–
LVEDD, mm (mean, ±SD)	4.8 (0.5)	–	–	–
Stroke volume, mL (mean, ±SD)	63.4 (16.2)	65.1 (22.1)	0.971	−0.1 (12.3)
Mean TPG, mmHg (mean, ±SD)	3.2 (1.4)	15.0 (3.4)	0.000	11.8 (3.2)
Peak TPG, mmHg (mean, ±SD)	7.0 (2.6)	25.9 (5.5)	0.000	18.9 (4.8)
SPAP, mmHg (mean, ±SD)	21.4 (3.8)	41.8 (8.9)	0.000	22.6 (8.3)

MV regurgitation severity (*n*, %)			0.500	
– None/trace/mild – ≥Moderate	19 (76)/6 (24)/0 (0)0 (0)	17 (68)/8 (32)/0 (0)0 (0)		– –

MV area, cm^2^ (mean, ±SD)	1.5 (0.3)	–	–	–

AoS severity (*n*, %)			0.006	
– None/mild – ≥Moderate – Unknown	13 (52)/5 (20) 0 (0) 7 (28)	12 (48)/5 (20) 1 (4) 7 (28)		– – –

AR severity (*n*, %)			1.000	
– None/trace/mild – ≥Moderate – Unknown	22 (88)/1 (4)/0 (0) 0 (0) 2 (8)	22 (88)/1 (4)/0 (0) 0 (0) 2 (8)		– – –

*SBP, systolic blood pressure; DBP, diastolic blood pressure; MAP, mean arterial pressure; LV, left ventricular; EF, ejection fraction; LVESD, left ventricular end-systolic dimension; LVEDD, left ventricular end-diastolic dimension; TPG, transmitral pressure gradient; SPAP, systolic pulmonary artery pressure; MV, mitral valve; AoS, aortic stenosis; AR, aortic regurgitation*.

### Peak Echocardiographic Data

**Figure 1:**
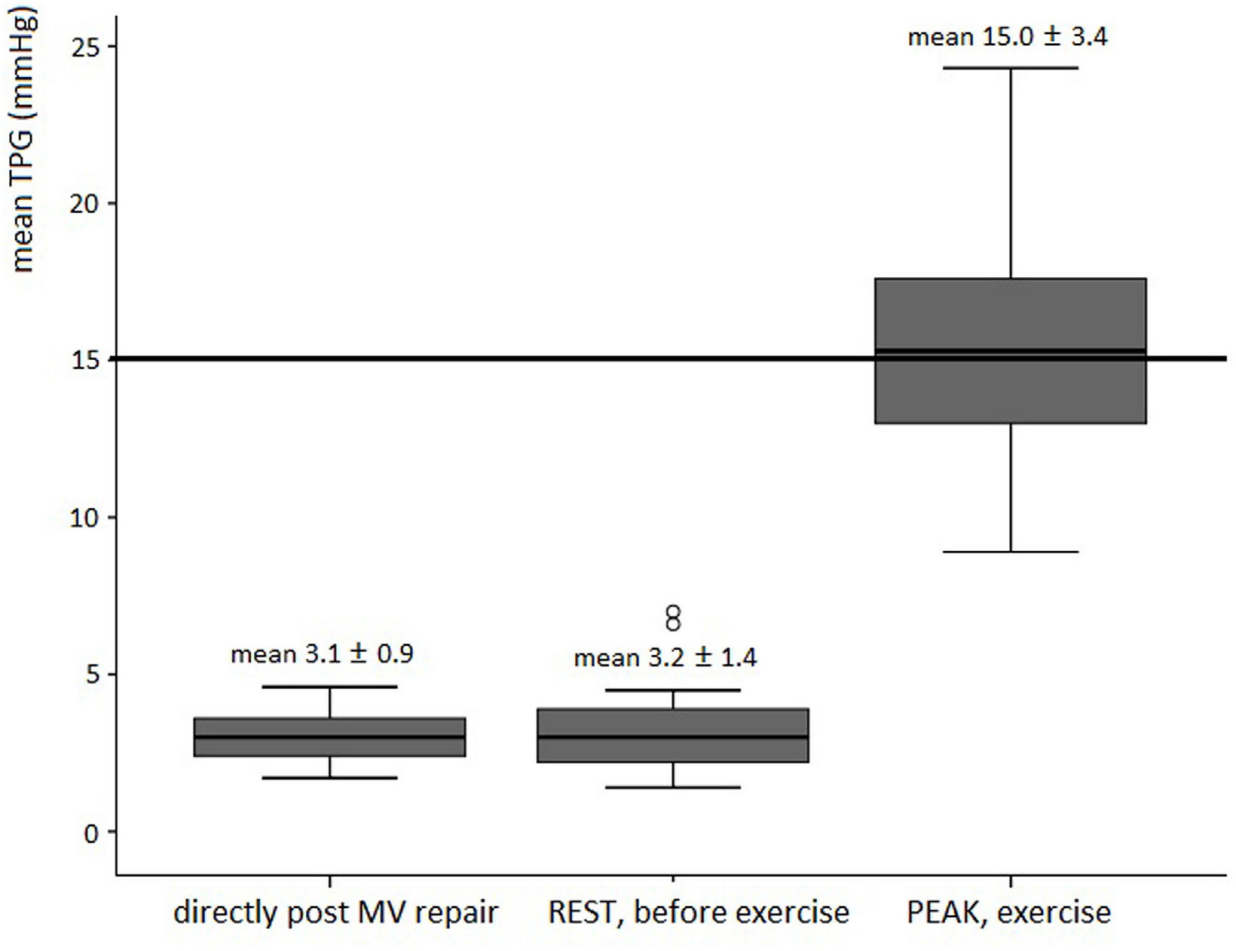
Boxplot figures showing the mean TPG directly post mitral valve repair, at rest (before stress echo) and peak exercise in asymptomatic patients post MV repair (*n* = 25). The reference line (thick line) is the current recommendation for intervention in native valve disease (3, 4). Abbreviation: TPG, transmitral pressure gradient; MV, mitral valve.

**Figure 2:**
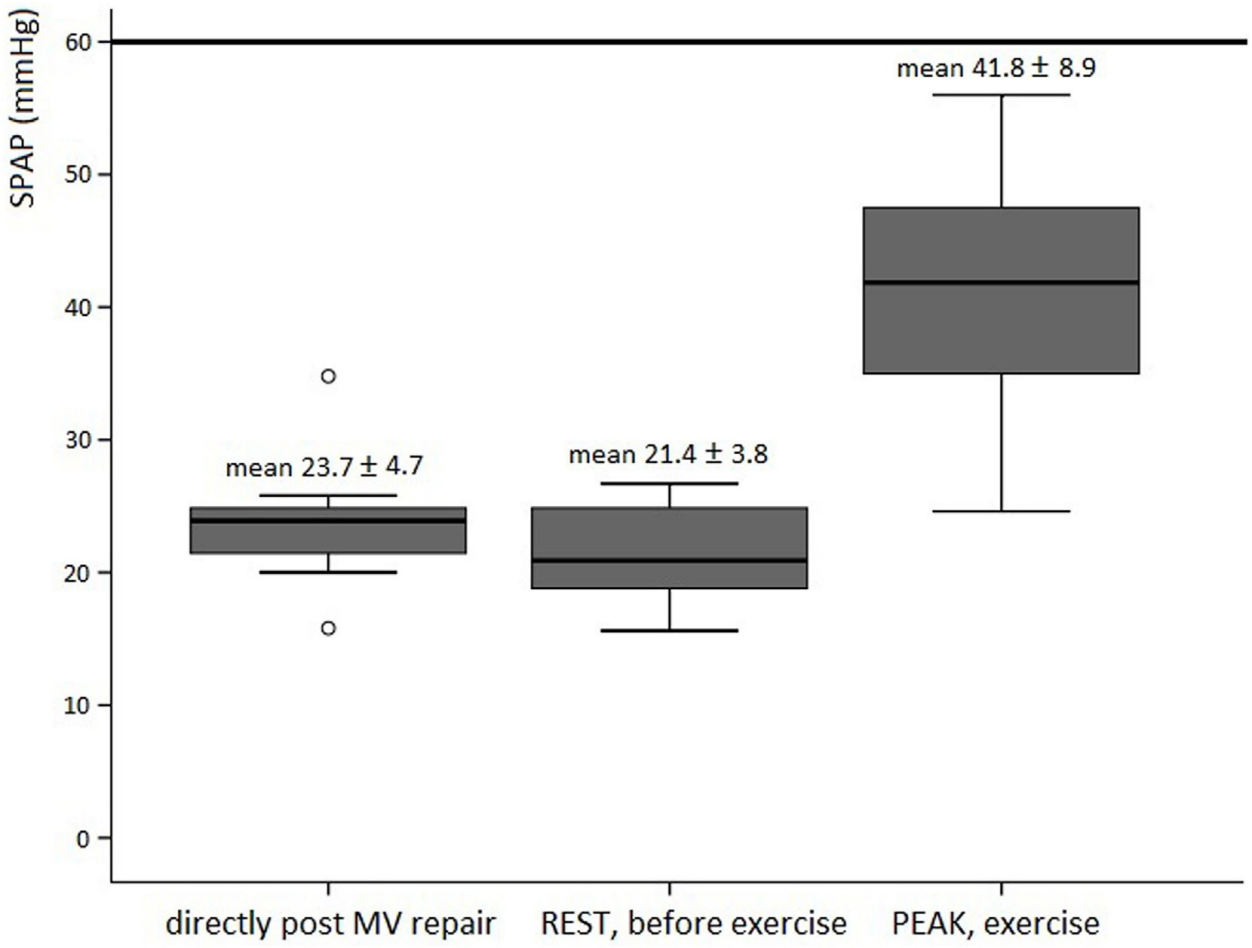
Boxplot figures showing the SPAP directly post MV repair (*n* = 11), at rest (*n* = 8), and peak exercise (*n* = 14) in asymptomatic patients post MV repair. The reference line (thick line) is the current recommendation for intervention in native valve disease (3, 4). Abbreviations: SPAP, systolic pulmonary artery pressure; MV, mitral valve.

Exercise characteristics are depicted in Table 3. Disproportionate shortness of breath, chest pain or arrhythmias did not occur. Supine exercise significantly increased heart rate and systemic blood pressure in all subjects. A rise in mean TPG and SPAP was significantly correlated to the increase in heart rate. Mean TPG markedly increased at peak exercise compared with the resting state (Figure 1). Fourteen patients (56%) reached a mean TPG at peak beyond the current recommendation for intervention in symptomatic native MV disease (>15 mmHg), with a maximum value of 24.3 mmHg. In all patients, the mean TPG more than doubled compared with the resting state. Peak SPAP was measured in 14 subjects. Although increasing during stress testing, SPAP at peak exercise remained within normal values in all patients (Figure 2). Only two patients showed an SPAP at peak of >50 mmHg, with a maximum value of 56.0 mmHg. An SPAP of >60 mmHg was never seen. Six patients terminated exercise before the 85% of the age-predicted heart rate limit; in two patients resulting from beta-blocker medication and in four patients because of leg exhaustion/complaints (muscular related).

**Table 3:** Exercise characteristics (*n* = 25).

	Total population
Maximal workload, W (mean, ±SD)	166 (29)
Maximal workload, patients per group (*n*, %)115 W 125 W 150 W 175 W 200 W 225 W	1 (4) 3 (12) 8 (32) 7 (28) 5 (20) 1 (4)
Exercise duration, min (mean, ±SD)	24 (5)
Peak exercise heart rate (mean, ±SD)	151 (15)
Reaching 85% of age-predicted HR (*n*, %)	19 (76)
Peak exercise blood pressure (mean, ±SD)	
– Systolic – Diastolic – MAP	204 (27) 92 (13) 130 (15)
Stopping reason (*n*, %)	
– (Leg) exhaustion – Angina pectoris – Arrhythmia – Other	25 (100) 0 (0) 0 (0) 0 (0)

*HR, heart rate; MAP, mean arterial pressure*.

### Subgroup Analyses Based on Mean TPG at Peak Exercise

Exploratory comparisons were performed for the 14 subjects (56%) with mean TPG at peak of >15 mmHg and the 11 patients (44%) with mean TPG ≤ 15 mmHg. The mean indexed MV annuloplasty ring diameter was significantly smaller (16.7 ± 1.6 versus 18.7 ± 2.1 mm, *P* = 0.009), and mean change in SPAP was significantly higher (29.1 ± 6.5 versus 16.2 ± 3.0 mmHg, *P* = 0.011) in patients with >15 mmHg mean TPG at peak. No other comparisons reached statistical significance. Univariable regression analysis for the continuous value of mean TPG at peak exercise (Online Resource 2A in Supplementary Material) showed a significant, positive correlation with *change in SPAP* (*r* = 0.312, *P* = 0.001), and a negative correlation with *indexed diameter MV ring diameter* (*r* = −0.968, *P* = 0.003). Mean TPG at peak only remained significantly correlated to the *indexed diameter of the MV ring annulus* (*r* = −1.089, *P* = 0.012) after correction for other variables and potential confounders.

### Subgroup Analyses Based on SPAP at Peak Exercise

Since no patients reached an SPAP > 60 mmHg, an exploratory comparison of subgroups with SPAP lower and above the guidelines reference value could not be performed. In a univariable analysis (Online Resource 2B in Supplementary Material), SPAP at peak exercise was significantly correlated to both *MV annulus ring type* and the *indexed diameter of the MV ring annulus* (*r* = −10.233, *P* = 0.026 and *r* = −2.568, *P* = 0.033, respectively). In a multivariable regression model correcting for other variables and potential confounders, no parameters remained independently correlated.

## Discussion

Our study concerning stress echocardiography in asymptomatic patients to identify the reference values after successful MV repair, revealed three major findings. Surprisingly, >50% of the patients showed a mean TPG at peak beyond the guideline recommendation (15 mmHg); however, mean TPG at peak was never >25 mmHg. Second, SPAP was always <57 mmHg at peak exercise, and therefore never reached the recommendation for intervention in native valve disease (>60 mmHg). Finally, only the indexed MV ring diameter remained independently correlated to the mean TPG at peak in a multivariable regression model.

### Changes in Mean TPG at Peak Exercise

The significant increase of mean TPG from 3.2 ± 1.4 mmHg at rest to 15.0 ± 3.4 mmHg at peak exercise in our study corresponds to previous literature (13, 27). The studies of Mesana et al. and Chan et al. showed for the first time changes in mean TPG after MV repair for myxomatous degeneration (13, 27). Similar to results in ischemic MV regurgitation (12, 14), elevated TPG after annuloplasty was not uncommon and related to higher NYHA class, worse intracardiac hemodynamics and poorer functional status. However, they included patients in NYHA ≥ II (39%), in contrast to our subjects that were all in NYHA 1. Asymptomatic patients with elevated TPG in our study revealed no reduction of exercise capacity; however, the exercise workload was even higher. This excellent exercise performance regardless higher mean TPG values were also shown previously (28) and might be explained by the flow-dependent characteristics of TPG. Patients with a higher EF and cardiac output create a higher mean TPG without negative impact on exercise capacity, whereas relative normal gradients in patients with reduced LV function may mask functional MV stenosis (28).

Elevated TPG during exercise is due to an obstruction that can occur at the mitral annulus and/or leaflet level (14, 29). The use of a complete MV ring might limit the annular excursion, partially explaining the restricted transmitral blood flow (30). In our study, patients with a complete ring had a significant smaller indexed MV ring diameter compared with patients with a Cosgrove Band, and mean TPG at peak exercise was significantly higher in subjects with smaller (<34 mm) compared with larger ring diameters (Online Resource 3 in Supplementary Material). However, MV ring type was not directly related to mean TPG at peak exercise. Previous studies confirm the correlation of a complete ring with a higher elevated TPG (13, 27). Also a mean TPG > 10 mmHg for all patients who received a ring annuloplasty <34 mm was seen, revealing annulus obstruction as a cause of elevated TPG (13). To this day studies have failed to demonstrate an effect of higher than expected TPG on clinical outcome (28, 31–33). Therefore, the impact of postoperative MV stenosis remains controversial.

### Changes in SPAP at Peak Exercise

Our study showed a significant increase in SPAP during exercise; however, values were always within normal ranges (3, 4). The change in SPAP at peak exercise was of more importance than the peak SPAP, as only a change in SPAP was strongly correlated to the mean TPG at peak. A similar trend was previously seen in 48 asymptomatic patients with significant MV stenosis: the increase in relative SPAP remained an independent determinant of the occurrence of dyspnea during exercise, whereas the cutoff value 60 mmHg could not discriminate patients with impaired functional capacity (34).

Exercise testing is the ideal way to evaluate symptoms in patients with mild-to-moderate MV stenosis (35). Annuloplasty, even in the absence of leaflet abnormalities or an undersized ring, can induce mechanisms and SPAP dynamics similarly to that in MV stenosis (12–14), leading to worse functional capacity. However, in our study, the exercise capacity was not decreased, nor related to SPAP at rest or peak exercise. Nevertheless, univariable analysis in our study did show a relation between higher pulmonary pressures at peak exercise and smaller complete MV rings, which has been described before (13).

### Other Modalities

The idea of early surgery in patients with MV regurgitation is to prevent occurrence of myocardial dysfunction preceding heart failure. Nevertheless, previous literature suggests unnoticed diastolic and systolic dysfunction due to myocardial stiffness and decreased fillings rates, as a result of enhanced LV filling and increased chamber compliance already in the early stages of MV regurgitation (36, 37). In addition, the valvular–ventricular interaction changes after MV surgery may influence LV function. However, when conventional echo parameters fail to detect potential subclinical damage (38), recurrent or persistent complaints after isolated MV repair remain a clinical challenge. Next to exercise echocardiography, as discussed in the current study, other modalities might be helpful.

Strain analysis has been used as a quantitative approach to estimate regional myocardial contractility and predict LV dysfunction. By using speckle tracking Goebel et al. revealed the impairment of regional myocardial deformation rate and LV systolic and diastolic twist rate, as proof of early myocardial damage in patients with arterial hypertension but still preserved EF (39). Candan et al. showed that (mainly basal) rotational parameters were significantly decreased after MV surgery; however, MV repair resulted in better rotational deformation parameters compared with replacement, possibly due to the preserved MV apparatus and therefore intact valvular–ventricular interaction (40). Larger follow-up studies are necessary to determine the clinical value of 2D speckle tracking echocardiography for prognostic assessment and guidance of therapy in patients with MV regurgitation.

Potential LV dysfunction in patients after MV surgery may lead to tissue deoxygenation, in particular during exercise. Decreased microvascular tissue perfusion has been demonstrated in patients with no global hemodynamic compromise or laboratory signs of hypoperfusion, using the new Sidestream Dark Field imaging technique (41). The assessment of the peripheral microcirculation as part of the initial evaluation in patients after MV surgery might be of additional value in clinical decision-making when symptoms occur.

Assessment of LV filling pressures in symptomatic patients after MV surgery may prove or refute a cardiac cause for the shortness of breath. Lower LV filling rates as a result from increased LV stiffness (increased LV diastolic pressures) can lead to lower MV gradients and therefore an underestimation of the postoperative severity of functional MS. Normal LV filling pressures in patients with normal MV gradients suggest a non-cardiac cause for the symptoms. In patients with preserved LV function, the ratio of early transmitral velocity to tissue Doppler mitral annular early diastolic velocity (*E*/*E*′) has been shown to correlate with LV filling pressures (42, 43); however, this correlation is influenced by MV regurgitation and MV surgery (44, 45). Goebel et al. therefore suggest to measure a combination of several Doppler parameters to determine LV filling pressures in these specific patient cohort. Unfortunately we did not incorporate LV filling pressures in current study analyses.

### Implications for Clinical Practice

Our study results implicate that guideline recommendations in native MV disease cannot be extrapolated to asymptomatic patients after successful MV repair: a mean TPG of 15 mmHg is not a clinically useful parameter after MV repair, as it does not identify those with a reduced exercise capacity and therefore cannot be used to guide therapy. Based on current series, the observation of symptoms and hemodynamic changes in SPAP during stress testing can be taken into account for decision-making process; however, future studies are needed to confirm this hypothesis. As long as guideline reference values after successful MV repair are lacking, a baseline stress echo assessment may be useful in the follow-up of these patients, while individual changes to a reference point rather than absolute values may be of more value for clinical decision-making when symptoms occur. For this purpose, stress echocardiography may be performed as soon as patients have recovered from surgery and finished the cardiac rehabilitation program successfully.

### Limitations

Twenty-five subjects were included from a larger series of patients after MV repair. This may have led to a selection bias. The sample size of our study was limited. Evaluation of 2D TTE in daily practice is limited by poor imaging windows, especially during exercise. Since we have no long-term follow-up data, the functional significance of more than expected increase in mean TPG after MV repair is still uncertain. A prospective study with longer follow-up is obligatory. Also a direct comparison between asymptomatic and symptomatic patients after MV repair is needed to confirm our results.

## Conclusion

Our data indicate that the standard value for mean TPG during stress echocardiography in asymptomatic patients after successful MV repair was above the guideline threshold of 15 mmHg in more than 50% of the patients. However, mean TPG at peak was always <25 mmHg. The maximum SPAP at peak exercise was 57 mmHg, hence never reaching the guideline recommendation for intervention (60 mmHg), although the change in SPAP during exercise may serve as an important parameter in case of symptoms. We think that baseline assessment of exercise derived echo parameters after MV repair may serve as an individual reference, and therefore could possibly be of additional value in clinical decision-making when symptoms occur. Future studies should clarify the relationship to adverse clinical outcomes in this cohort.

## Ethics Statement

The protocol was approved by the “Research Ethics Board” of the UMC Utrecht (file number NL39865.041.14; protocol number 14-483). All subjects gave written informed consent in accordance with the Declaration of Helsinki.

## Author Contributions

All authors (RJ, KU, MC, EA, PZ, PD, JK, and SC) substantially contributed to the conception and design of this work, the analyses and interpretation of the data; critically revised this work for important intellectual content; approved the final version to be published; and agreed to be accountable for all aspects of the work in ensuring that questions related to the accuracy or integrity of any part of the work are appropriately investigated and resolved.

## Conflict of Interest Statement

The authors declare that the research was conducted in the absence of any commercial or financial relationships that could be construed as a potential conflict of interest.
