# Supplementary material for: Reference Values for Physical Stress Echocardiography in Asymptomatic Patients after Mitral Valve Repair
Source: Front Surg. 2018 Feb 19;5:6. doi: 10.3389/fsurg.2018.00006 (PMC5826059; doi:10.3389/fsurg.2018.00006)

**Supplementary Material**

*Article title*: Reference values for physical stress echocardiography in asymptomatic patients after mitral valve repair

*Journal name*: Frontiers in Surgery – Heart Surgery

*Author names*: R. Jansen, K. Urgel, M.J. Cramer, E.E.H.L. van Aarnhem. P.P.M. Zwetsloot, P.A. Doevendans, J. Kluin, S.A.J. Chamuleau

*Contact details*

*Corresponding author*: Prof dr. S.A.J. Chamuleau, department of Cardiology University Medical Center Utrecht. Heidelberglaan 100, 3584 CX UTRECHT
 E-mail: s.a.j.chamuleau@umcutrecht.nl; Tel. +31 88 75 67903

This document includes Online Resource 1, 2A and 2B, and 3.

ONLINE RESOURCE 1.

Flowchart of the study design

*2D: two-dimensional, TTE: transthoracic echocardiogram*

*
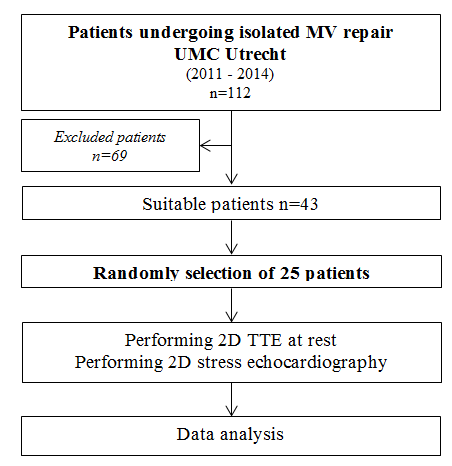
*

ONLINE RESOURCE 2.

A. Linear regression: univariable and multivariable analysis in mean TPG at peak exercise using the continues values

|  | **Univariable analysis** |  | **Multivariable analysis*** |  |
| --- | --- | --- | --- | --- |
| **Variables** | **Β** | **P-value** | **Β** | **P-value** |
| Change in SPAP (n=8) | 0.312 | ***0.001*** | ** | ** |
| MV ring type (n=25) | -2.347 | 0.095 | -1.639 | 0.369 |
| Indexed MV ring diameter (n=25) | -0.968 | ***0.003*** | -1.089 | ***0.012*** |
| Surgical resection performed (n=25) | 1.430 | 0.312 | 1.427 | 0.350 |
| *SPAP: systolic pulmonary artery pressure, MV: mitral valve*  ** Based on n=20; corrected for: age, gender, mitral valve area at rest, mitral valve regurgitation grade at peak exercise, heart rate at peak exercise, ejection fraction at rest, total months after mitral valve repair.*  *** Although strongly correlated to mean transmitral pressure gradient at peak, we did not include change in systolic pulmonary artery pressure in the multivariable model due to the small number of measurements.* | | | | |

ONLINE RESOURCE 2.

B. Linear regression: univariable and multivariable analysis in SPAP at peak exercise using the continues values

|  | **Univariable analysis** |  | **Multivariable analysis*** |  |
| --- | --- | --- | --- | --- |
| **Variables** | **B** | **P-value** | **Β** | **P-value** |
| Mean TPG at peak (n=14) | 1.599 | 0.070 | 0.189 | 0.925 |
| MV ring type (n=14) | -10.233 | ***0.026*** | -10.229 | 0.353 |
| Indexed MV ring diameter (n=14) | -2.568 | ***0.033*** | -1.985 | 0.471 |
| Surgical resection performed (n=14) | 3.117 | 0.538 | -0.404 | 0.974 |
| *TPG: transmitral pressure gradient, MV: mitral valve*  ** Based on n=12; corrected for: age, gender, mitral valve area at rest, mitral valve regurgitation grade at peak exercise, heart rate at peak exercise, ejection fraction at rest, total months after mitral valve repair.* | | | | |

ONLINE RESOURCE 3.

Scatterplot showing a significant relation (P=0.001) between mean TPG at peak and MV ring diameter in asymptomatic patients after successful MV repair with a Carpentier-Edwards Physio ring (n=15), and no significant relation (P=0.246) between mean TPG at peak and MV ring diameter in asymptomatic patients after successful MV repair with a Cosgrove-Edwards band (n=10).

*TPG: transmitral pressure gradient, MV: mitral valve*


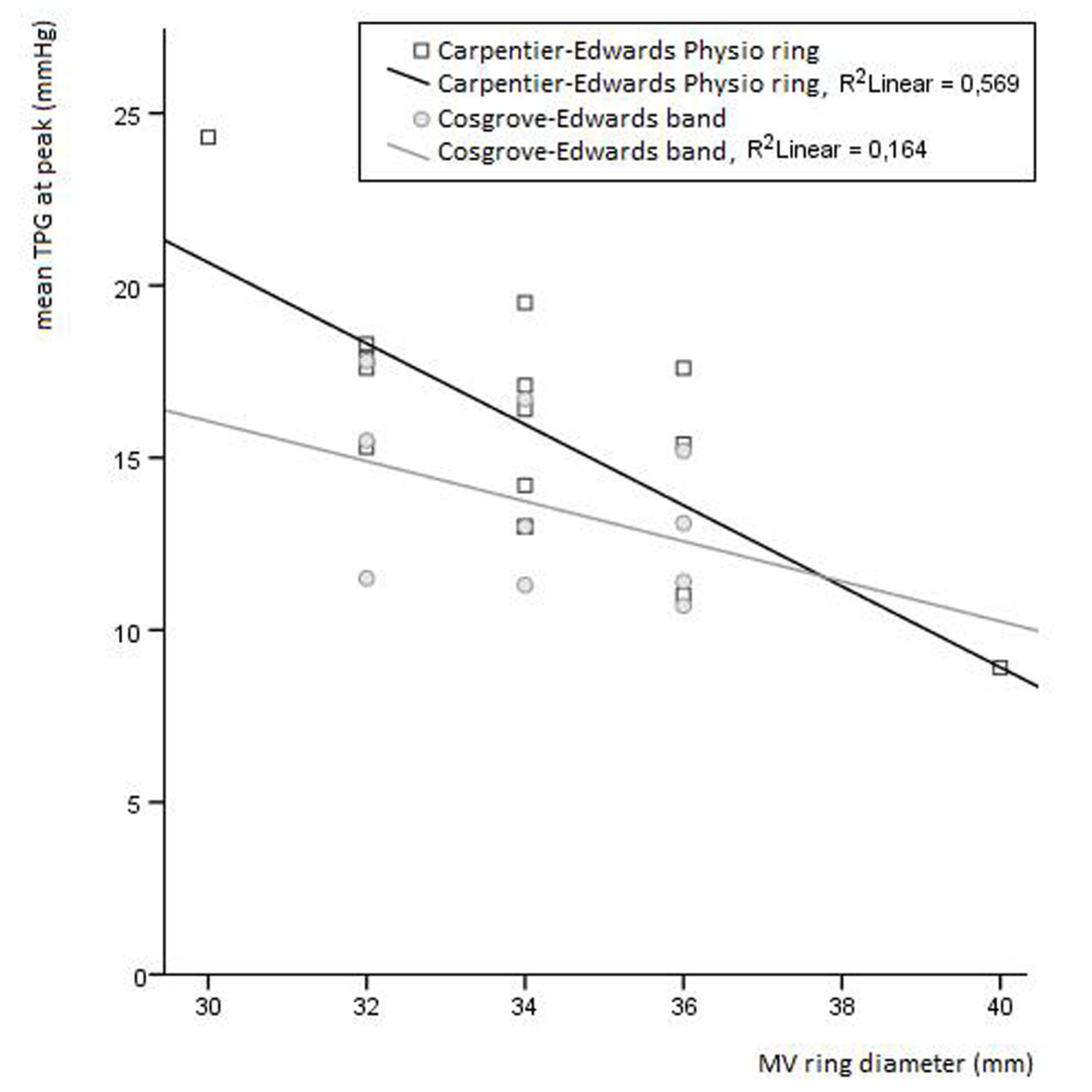

Supplement: Supplementary file 1 [file Data_Sheet_1.DOCX]
